# Temperature-dependent Raman spectroscopy in double spiral WS_2_ nanostructures

**DOI:** 10.1039/d5ra03857j

**Published:** 2025-10-15

**Authors:** Jing-Ming He, Jie Gao, Chun-Guang Huo, Min-Ru Qi, Xiang-Dong Li, Shu-Hui Hu, Yu-Jun Shi, Shen Wang, Xiao-Peng Fan, Cheng-Bing Qin

**Affiliations:** a Sanli Honors College, School of Physics and Electronics Engineering, Shanxi University Taiyuan China; b School of Physics and Electronics Engineering, Shanxi University Taiyuan China; c Institute of Laser Spectroscopy, Shanxi University Taiyuan China chbqin@sxu.edu.cn; d Institute of Theoretical Physics, Shanxi University Taiyuan China; e College of Physics and Optoelectronic Engineering, Taiyuan University of Technology Taiyuan 030024 China

## Abstract

The exploration of complex morphological hierarchies in two-dimensional transition metal dichalcogenides (TMDs), such as spiral structures, is critical for advancing their applications in phonon engineering and nano thermites. However, the intricate thermal properties of multi-spiral architectures, which are expected to exhibit more complex strain fields and interlayer interactions, remain largely unexplored. Addressing this gap, this study presents a temperature-dependent Raman spectroscopy investigation on a double-spiral WS_2_ structure over the range of 150–450 K. Using multi-peak Lorentzian fitting, the first-order optical modes E^1^_2g_ and A_1g_ were successfully extracted. The results show that, relative to the monolayer and single-spiral structures, the double-spiral WS_2_ has a weaker temperature response. Layers within the same spiral domain show similar temperature dependent Raman shift behaviors, whereas those from different spirals display distinct trends. Fitting with thermal expansion and multiphonon models reveals that the nonlinear temperature dependence is primarily governed by thermal expansion, which can be directly described by the thermal expansion coefficient. And the three-phonon process dominating the shift magnitude—except in the edge layer. Furthermore, the analysis suggests potential evidence consistent with the theoretically predicted negative thermal expansion effect in WS_2_, which merits further investigation.

## Abstract 2

Based on temperature-dependent Raman spectroscopy of double spiral WS_2_, this work reveals layer-specific thermal responses dominated by thermal expansion and provides experimental evidence of negative thermal expansion.

## Graphical abstract

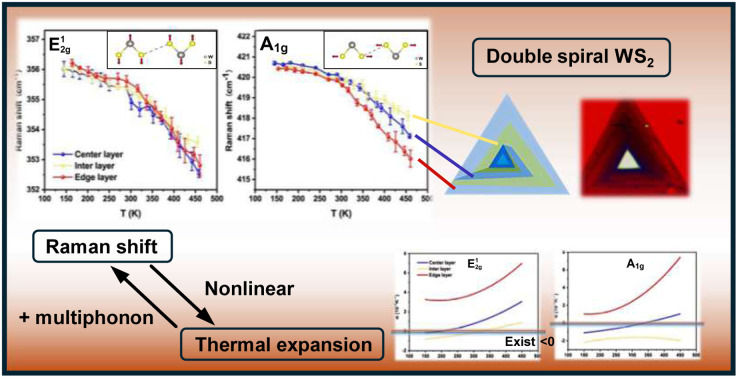

## 1 Introduction

**Fig. 1:**
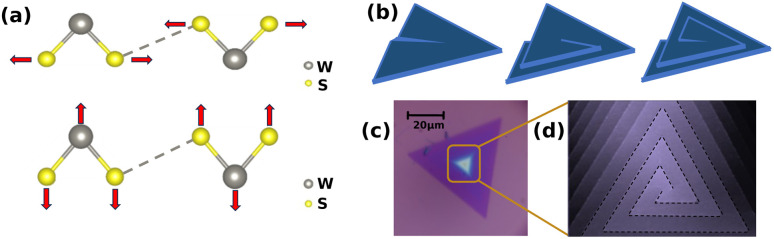
(a) Vibrational schematics of two optical modes in WS_2_. The upper image illustrates A_1g_ mode, while the lower image depicts the E^1^_2g_ mode. Dashed lines represent weak interlayer van der Waals interactions. (b) Schematic growth model of a single spiral WS_2_ structure driven by screw dislocation. (c) Optical image of double spiral WS_2_ structures. (d) Schematic illustration of a double spiral configuration.

Since the successful mechanical exfoliation of graphene in 2004, the field of two-dimensional (2D) materials has witnessed remarkable progress in synthesis methods, structural and property investigations, as well as in the development of innovative applications.^1–18^ Among various 2D materials, transition metal dichalcogenides (TMDs) have garnered considerable interest due to their remarkable properties and diverse potential applications.^19,20^ In particular, tungsten disulfide (WS_2_), a representative TMD material, consists of a tungsten (W) atomic layer sandwiched between two sulfur (S) atomic layers, forming an S–W–S “sandwich” structure. In Raman spectroscopy, its E^1^_2g_ vibration mode (∼355 cm^−1^) corresponds to the in-plane cooperative vibration of W and S atoms, while the A_1g_ mode (∼419 cm^−1^) originates from the out-of-plane vibration of S atoms along the interlayer direction, as showed in Fig. 1(a). Furthermore, the atomic layer structure of WS_2_ exhibits excellent mechanical flexibility and chemical stability, which shows great application potential in fields such as flexible electronic devices and optoelectronic devices^21–24^

In recent years, WS_2_ with spiral structures has attracted significant research attention. These unique configurations, arising from interlayer misorientation and twisting during crystal growth,^25,26^ have been recognized as a research hotspot, and their potential in advanced applications such as thermal management and phonon engineering has also been highlighted.^27–29^ The formation of spiral WS_2_ is closely related to specific growth mechanisms, typically realized through a screw dislocation-driven (SDD) process.^30^ This mechanism initiates under low supersaturation conditions, where screw dislocation line defects serve as a persistent step source, guiding the continuous attachment of precursors and the upward spiral propagation of layers until spatial confinement terminates the process, resulting in a distinct helical geometry (Fig. 1(b)). The SDD growth not only ensures crystallographic orientation consistency between successive layers, forming stacked edges with atomic-level registry, but also induces continuous twisting and interlayer strain distributions that may lead to phonon dynamics and interlayer coupling behaviors markedly different from those of conventional layered WS_2_.^25,31,32^

While notable progress has been achieved in the controllable synthesis and fundamental property studies of spiral WS_2_,^30,33–35^ a deeper understanding of its fundamental physics—particularly the evolution of phonon scattering and lattice dynamics with temperature—remains crucial. In real device operation, inevitable temperature variations can significantly affect physical properties such as electrical and thermal conductivity. Although some studies have reported nonlinear temperature responses in single-spiral WS_2_,^36^ a fundamental understanding of the thermal properties of multi-spiral systems is entirely lacking. Such structures pose intriguing questions: How does the interaction between adjacent spirals influence the overall thermal response? Do layers belonging to different spiral domains behave differently? Addressing these questions is critical for moving from fundamental understanding to the practical design of devices based on these complex morphologies. The present work aims to address this critical gap by extending these investigations to a double-spiral WS_2_ structure, which provides an ideal model system to unveil the effects of inter-spiral coupling on phonon dynamics. We anticipate more complex internal strain fields and interlayer interaction differences,^37^ thereby significantly enriching the understanding of structure–property relationships in spiral TMDs.

Double spiral WS_2_, in this study, moriginates from an initial crystal nucleus formed by two adjacent screw dislocation cores with the same handedness, resulting in two spiral terraces that propagate outward in parallel, as shown in Fig. 1(c) and (d). Such a geometry provides an ideal model to reveal potential non-uniform responses to thermal perturbations arising from inter-spiral coupling. Raman spectroscopy, owing to its capability for directly and sensitively probing the temperature evolution of lattice vibrations (phonon modes), is a powerful tool in this context. By mapping and analyzing the temperature dependence of the Raman shifts of the E^1^_2g_ and A_1g_ modes, the center and edge layers within the same spiral domain show nearly identical temperature dependent Raman shift trends, while the inter layer from the other spiral domain displays a comparatively smoother thermal response. Further fitting using thermal expansion and multiphonon interaction models revealed that the nonlinear temperature response is primarily governed by thermal expansion, with the three-phonon process determining the magnitude of the shift (except for the edge layer, whose volume exhibits a more sensitive response to temperature). Additionally, this work presents possible experimental evidence for the theoretically predicted negative thermal expansion effect in WS_2_.

## 2 Experimental setup and Raman spectroscopy

**Fig. 2:**
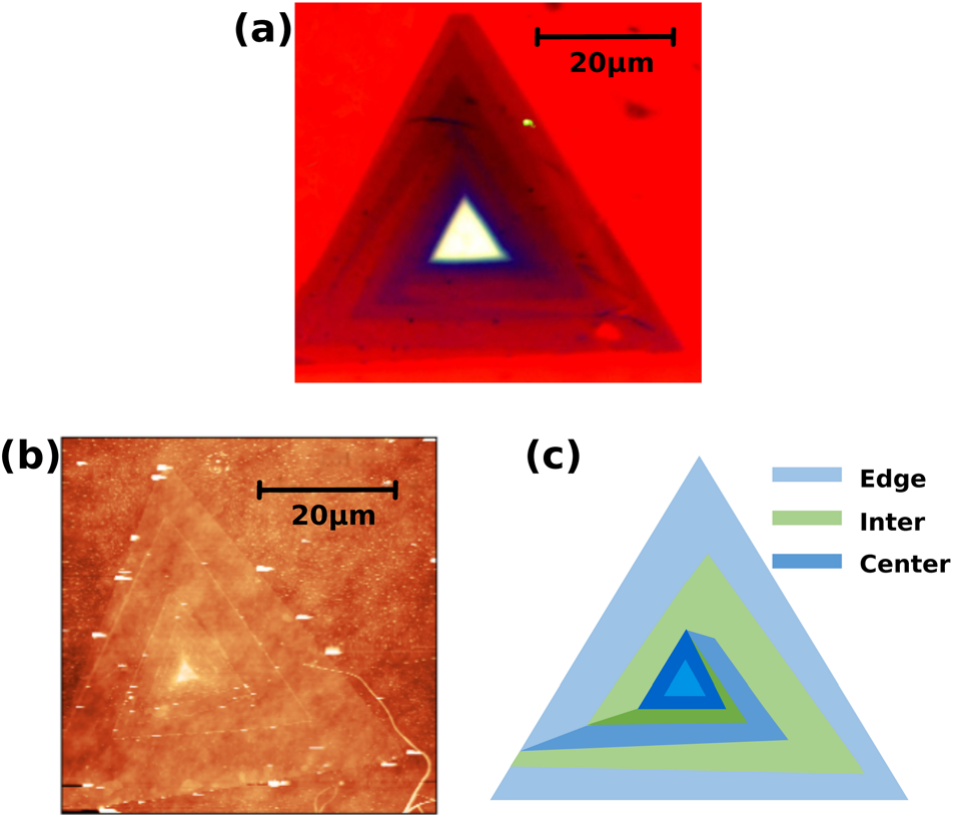
(a) Optical microscopy image of the sample. Only the red channel is shown to enhance the contrast between different layers. (b) The AFM image of the sample, where the white line corresponds to the layer boundary. (c) Schematic diagram of the double spiral structure of the sample. The three measured layers, labeled from outside to inside, are referred to as the edge layer, the inter layer, and the center layer, in which the edge and center layers belong to the same spiral domain.

Well-defined WS_2_ samples with distinct morphological hierarchy were specifically selected for micro-Raman mapping analysis, as illustrated in Fig. 2(a). Spiral WS_2_ were produced by chemical vapor deposition (CVD) on a silicon substrate using SDD growth techniques, the details of which can be found in ref. 30. Extracting a single color from an image for observation is effective for distinguishing regions occupied by different layers in the experiment. The red part is selected here. The layered structure of the sample can be accurately characterized by atomic force microscopy (AFM), as shown in Fig. 2(b), where each fine bright white line corresponds to an individual layer within the spiral structure. AFM results reveal that the center layer, near the core region of the spiral dislocation, exhibits slight interlayer stacking features due to rotational misalignment and stacking-sequence variations. In contrast, the edge and inter layers display typical epitaxial monolayer characteristics. Collectively, these AFM results confirm the structural identity of each layer and provide strong support for the regional assignments in the Raman measurements of this study.

A more intuitive schematic of the double-spiral configuration is provided in Fig. 2(c). In this study, the lattice dynamical properties of the three outer layers of the sample (center, inter, and edge layers) were investigated. The center and edge layers belong to the same spiral domain, being connected through a shared screw dislocation core. In contrast, the interlayer and the center layer belong to two independent spiral domains, each growing around a different dislocation core. In Fig. 2(c), each layer is represented by a distinct color, and all layers belonging to the same spiral domain are depicted in shades of the same color scheme.

This experiment uses a confocal Raman spectrometer (LabRAM HR Evolution) combined with a 532 nm laser to generate and record the Raman spectra of the material. The use of temperature-dependent Raman spectroscopy in this study was selected for its unique ability to probe layer-specific and domain-specific phonon behavior with high spatial resolution. It should be noted that techniques capable of providing direct structural evidence for thermal expansion, such as variable-temperature X-ray diffraction (XRD) or transmission electron microscopy (TEM), are not currently accessible within our experimental platform for this specific sample system. Consequently, this work aims to establish a robust phenomenological foundation for the thermal response of double-spiral WS_2_ using the most advanced and accessible methodology available to us, while explicitly identifying the requirement for future direct validation using more specialized instrumentation. In addition, the temperature dependent measurements employed two thermal control protocols: (1) a cryostat system (Dongfang Chenjing Liquid Nitrogen thermostat) (80–300 K range, ±2.0 K stability) utilizing liquid nitrogen cooling, and (2) a resistive heating stage (300–500 K range, ±4.0 K stability) for elevated temperature studies.

**Fig. 3:**
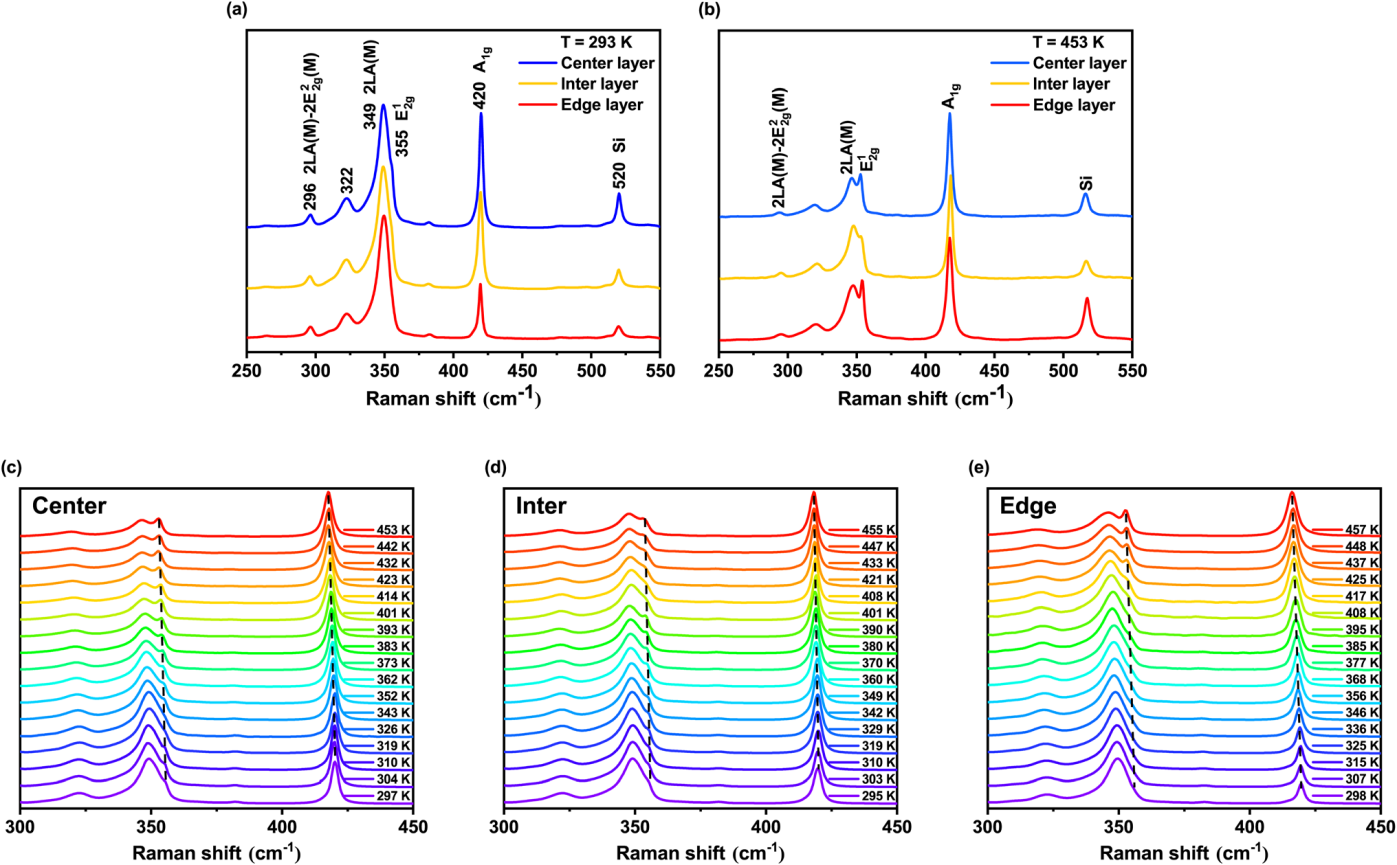
(a and b) Raman spectra of the outer three layers of the sample at 293 K and 453 K in the range of 200–500 cm^−1^. The blue line represents the spectrum of the center layer, the yellow line corresponds to the inter layer, and the red line represents the edge layer. (c–e) Temperature dependent Raman shifts of the E^1^_2g_ and A_1g_ modes for the center layer (c), inter layer (d), and edge layer (e), respectively. In each plot, the temperature increases from bottom to top. Dashed lines trace the positions of the E^1^_2g_ and A_1g_ peaks at each temperature, revealing their temperature dependent behavior.

The measured Raman spectra of the material are shown in Fig. 3(a) and (b), which displays the spectra of the outer three layers of the sample at room temperature (293 K) and high temperature (453 K). At high temperatures, within the range of 250–450 cm^−1^, the Raman spectra are dominated by first-order Raman processes, primarily corresponding to two optical vibrational modes: E^1^_2g_ ≈ 355 cm^−1^ and A_1g_ ≈ 420 cm^−1^. At room temperatures, second-order Raman effects are enhanced. The positions of several prominent second-order Raman peaks are marked in Fig. 3(a), among which the strongest is located at 2LA(M) ≈ 349 cm^−1^.

Considering the temperature dependence of E^1^_2g_ and A_1g_ modes, we present the Raman spectra in the temperature range of 295–457 K to investigate the overall temperature dependent trends, as shown in Fig. 3(c)–(e) a slight redshift in peak position is observed with increasing temperature—similar to the behavior reported for monolayer WS_2_ in previous studies.^38^ To further analyze the physical mechanisms underlying this phenomenon, it is necessary to extract precise peak parameters. It is worth noting that, under low-temperature conditions, the intensity of the second-order Raman peak 2LA(M) significantly exceeds that of E^1^_2g_, resulting in an overlap between the two peaks. This issue can be effectively resolved by employing a combination of second-derivative peak detection, multi-peak Lorentzian fitting, and first-order derivative-assisted Lorentzian fitting to achieve accurate separation of the overlapping peaks.

## 3 Variable temperature spectral analysis

Spectral deconvolution rigorously accounted for natural linewidth broadening mechanisms through Lorentzian line shape fitting, whose expression is shown by1
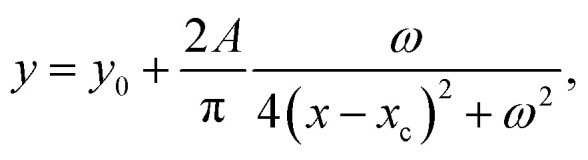
where *y*_0_ corresponds to the intensity of the baseline, generally, *y*_0_ = 0 after data preprocessing, *A* denotes the peak amplitude, *ω* corresponds to the full width at half maximum (FWHM), and *x*_c_ represents the Raman shift position.

### 3.1 The Raman shift analysis of spiral WS_2_ under variable temperature

**Fig. 4:**
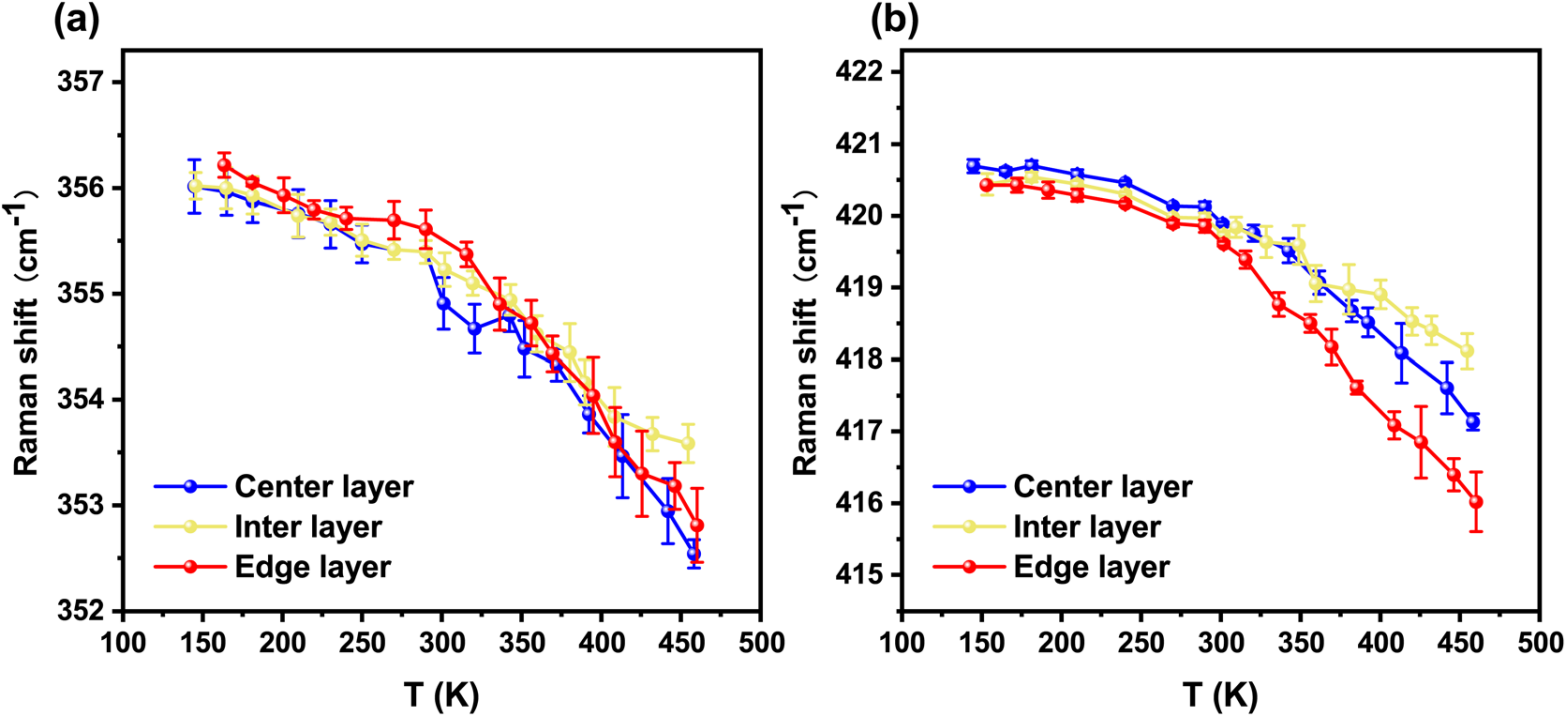
(a) Temperature-dependent Raman shifts of the E^1^_2g_ mode for the outer three layers of the sample. (b) Temperature-dependent Raman shifts of the A_1g_ mode for the outer three layers of the sample. In each plot, the blue line represents the center layer, the yellow line corresponds to the inter layer, and the red line denotes the edge layer.

As shown in Fig. 4, by applying the first-order derivative Lorentzian fitting method described in the previous section, the temperature-dependent Raman shifts of E^1^_2g_ and A_1g_ mode can be accurately extracted.

Fig. 4(a) and (b) clearly demonstrate the general temperature dependent trends of E^1^_2g_ and A_1g_ mode, exhibiting a weak temperature dependence(∼1 cm^−1^ per 100 K). From the spectral images, the Raman shifts of both the E^1^_2g_ and A_1g_ modes in each layer of the sample exhibit a gradual red shift with increasing temperature. Quantitatively, a linear model was employed to fit the data. Specifically, the first-order temperature-dependent properties were analyzed using

2 *ω*(*T*) = *ω*_0_ + *kT*,

where intercept *ω*_0_ (cm^−1^) is the Raman shift of the characteristic peak at absolute zero temperature, and slope *k* (cm^−1^ K^−1^) is the first order temperature coefficient. *ω*_0_ and *k* obtained for both modes in each layer are summarized in Table 1. We consider the full temperature range fit (*i.e.* Full-T fit, whose range is 150–450 K), compared with the linear coefficients reported in other literature. Compared with E^1^_2g_ mode of the monolayer WS_2_ (−0.0091 cm^−1^ K^−1^),^38^ the *k* of the inter and edge layers are smaller, whereas that of the center layer increases. In contrast, for A_1g_ mode one (−0.0121 cm^−1^ K^−1^),^38^*k* in the double-spiral WS_2_ are generally smaller. Even when compared with the single-spiral A_1g_ mode (−0.00896 cm^−1^ K^−1^ for the edge layer, −0.00901 cm^−1^ K^−1^ for the inter layer, and −0.00971 cm^−1^ K^−1^ for the center layer),^36^ the corresponding layers in the double-spiral WS_2_ still exhibit smaller slope.

**Table 1:** Linear fitting parameters of the E^1^_2g_ and A_1g_ modes at different temperature ranges

Mode/position	Full-*T* fit			Low-*T* fit			High-*T* fit		
	*ω*_0_	*k*	adjusted *R*^2^	*ω*_0_	*k*	adjusted *R*^2^	*ω*_0_	*k*	adjusted *R*^2^
E center	358.38	−0.01152	0.890	356.80	−0.00514	0.853	360.86	−0.01799	0.963
E inter	357.49	−0.00800	0.918	356.78	−0.00494	0.973	359.06	−0.01231	0.976
E edge	357.67	−0.00863	0.898	356.97	−0.00508	0.961	360.73	−0.01708	0.985
A center	422.42	−0.00855	0.775	421.73	−0.00590	0.883	426.44	−0.02029	0.998
A inter	422.14	−0.00798	0.928	421.96	−0.00730	0.900	423.58	−0.01197	0.950
A edge	421.47	−0.00679	0.784	421.15	−0.00473	0.942	426.66	−0.02329	0.979

This indicates that, as temperature increases, the stress variations induced in the spiral structure partially weaken the material's temperature dependence. Furthermore, in the double-spiral structure, the correlation between internal stress changes and interlayer coupling is weaker than in the single-spiral case, and these two effects together further reduce the material's dependence on temperature. Furthermore, it can be observed that the temperature-dependent trends of both modes are not strictly linear. More prominently, by separately fitting the low-temperature (*i.e.* Low-T fit, whose range is 150–300 K) and high-temperature (*i.e.* High-T fit, whose range is 300–450 K) ranges, the resulting fitting parameters are listed in Table 1. Such as the E^1^_2g_ mode of the center layer, it exhibits a slope of *k* = −0.00514 cm^−1^ K^−1^ at low temperature, which increases to *k* = −0.01799 cm^−1^ K^−1^ at high temperature, indicating a pronounced steepening trend. Similar behavior is also observed for the other modes.

In addition, for the E^1^_2g_ mode, the center and edge layers nearly identical trends, whose *k* is very similar at both low and high temperatures (the difference is within 0.001 cm^−1^ K^−1^). As for the inter layer, its Raman shift curve is also highly consistent with the previous two, albeit with slightly smoother variations. As can be seen from the parameters of the linear fitting (Table 1), under low-temperature conditions, *k* of the inter is comparable to that of the other layers, whereas under high-temperature conditions, it is significantly smaller (the difference is about 0.005 cm^−1^ K^−1^).

For A_1g_ mode, the analysis of the variation curves shown in Fig. 4(b) reveals that under low-temperature conditions, the three curves exhibit nearly identical trends. Things began to change under high temperatures. The redshift in the center layer gradually becomes weaker than that in the edge layer. The inter layer displays a smoother variation with temperature compared to the center and edge layers, particularly above 380 K. Similar to the E^1^_2g_ mode, this can be quantitatively described by comparing the fitting parameters (Table 1), where the *k* of the inter layer is approximately (−0.009 cm^−1^ K^−1^) smaller than that of the central and edge layers.

It can be observed that the temperature dependence of the modes in the inter and edge layer (or center layer) shows significant differences corresponding to the two different spiral domains. For the same spiral domain (*i.e.* the edge and center layer), considering that the helical geometry of spiral structures may induce compressive stress in inner layers and tensile stress in outer layers, which in turn can lead to noticeable differences between inner and outer layers,^39^ this to some extent explains the difference between the two layers of A_1g_ at high temperatures. And the absence of such discrepancies for E^1^_2g_ suggests that, in the case of double spiral configurations, possibly, the interlayer stress and the structural stress induced by the helicity are well balanced which results in nearly identical temperature-dependent behavior of the E^1^_2g_ mode across all layers.

It is worth noting that inflection points appear in the temperature-dependent Raman shift curves of edge layer in this range. A similar phenomenon was reported in ref. 38 for monolayer WS_2_, where the authors found that, similar to MoS_2_, wrinkles in the sample material led to weakened coupling with the substrate, resulting in the observed inflection points. In this study, since the double spiral samples used in the experiments have physical contact between the edge layer and the substrate, wrinkles in the material further reduce the substrate coupling, which in turn causes the inflection behavior observed in the temperature dependent Raman shift curves of A_1g_ mode. In contrast, the center (inter) layer—being farther from the substrate—does not exhibit such (obvious) inflection points. Following the approach adopted in ref. 38, only data below 380 K for the edge is considered in subsequent analyses.

To further investigate the nonlinear behavior of the two optical modes E^1^_2g_ and A_1g_, their data were fitted using a thermal expansion model combined with a multiphonon interaction model. Specifically, for the multiphonon contribution, only the effects of three-phonon and four-phonon processes were considered.

### 3.2 Consideration of thermal expansion model and multiphonon process

Considering the volumetric effect of spiral WS_2_ and the anharmonic effects in the material, temperature both indirectly affects the Raman shift of characteristic peaks through volume changes, and directly affects it. The multiphonon process mainly focuses on the three-phonon and four-phonon one, and there is a mapping relationship between temperature and Raman shift:

3 *ω*(*T*) = *ω*_0_ + Δ*ω*_V_ + Δ*ω*_T_,

where Δ*ω*_V_ corresponds to the change in Raman shift due to the volumetric effect, and Δ*ω*_T_ corresponds to the change due to the pure temperature effect.

Δ*ω*_V_ is often described using a thermal expansion model with the Grüneisen constant,^40^ as follows:4
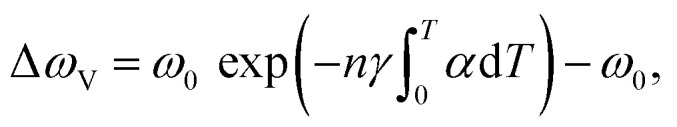
where the thermal expansion effect is related to the Raman shift at absolute zero temperature. *n* is the degeneracy, 1 for A_1g_ mode and 2 for E^1^_2g_ mode. *γ* is the Grüneisen constant. *α* is the thermal expansion coefficient, a function of temperature *T*. Since the Raman shift change with temperature is small, we expand *γα* and retain up to the second-order term:^38^

5 *γα* = *a*_0_ + *a*_1_*T* + *a*_2_*T*^2^.

The corresponding thermal expansion effect is:6
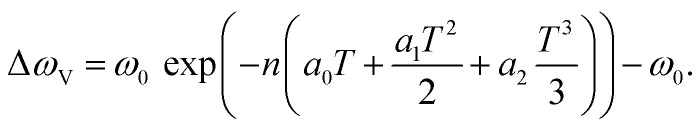


Δ*ω*_T_ is often described using phonons. Here, we focus on the anharmonic processes of the three-phonon and four-phonon interactions in the phonon–phonon process,^41^ whose specific form is as follows:7
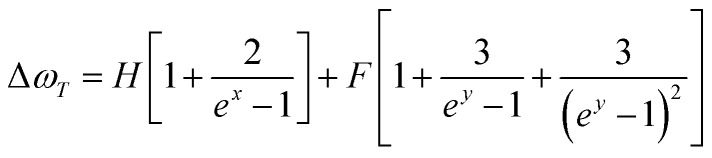
The first term represents the three-phonon process, and the second term represents the four-phonon process. *x* = *hω*/2*kT*, *y* = *hω*/3*kT*, where *ω* corresponds to the phonon frequency. Here, for convenience of fitting, the variation of the Raman shift with temperature is treated as a small quantity, and the first-order approximation of *ω* is taken as *ω*_0_.

Regarding the parameter *ω*_0_, considering the weak temperature dependence presented in Fig. 4, the value of *ω*_0_ is determined from the intercept parameter obtained by linear fitting of the data. It should be noted, however, that the temperature-dependent curves of the Raman shifts for both modes exhibit nonlinear effects. To minimize the bias introduced by these effects, the intercept parameter obtained from the linear fitting of the low-temperature data is adopted as the value of *ω*_0_.

**Fig. 5:**
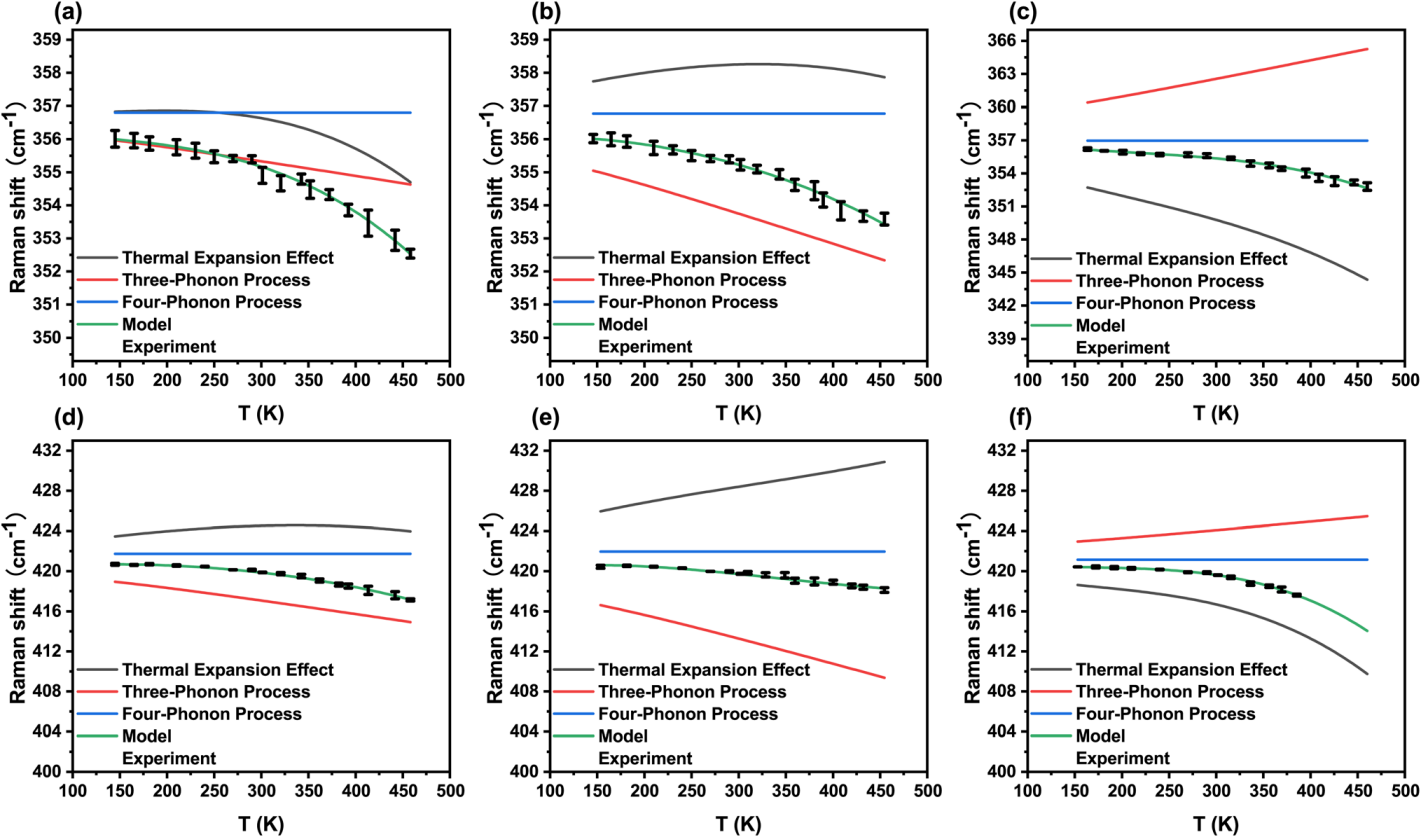
(a–c) Model fitting results for the temperature dependent Raman shifts of the E^1^_2g_ mode in the outer three layers of the sample: (a) center layer, (b) inter layer, and (c) edge layer. (d–f) Model fitting results for the A_1g_ mode in the corresponding layers: (d) center layer, (e) inter layer, and (f) edge layer. In all plots, scatter points represent experimental data. The black solid line denotes the contribution from thermal expansion, the red solid line corresponds to the three-phonon process, the blue solid line represents the four-phonon process, and the green solid line represents the full model.

The temperature dependent Raman shift fitting results for the two optical modes, E^1^_2g_ and A_1g_, along with the corresponding fitting parameters, are summarized in Fig. 5, Tables 2 and 3. During the fitting process, it was found that the use of only the three-phonon process was sufficient to accurately reproduce the experimental curves. When the optimized weight *H* for the three-phonon term was fixed, the resulting value of *F* for the four-phonon term was found to be negligibly small. Therefore, *F* = 0 was used in the final fitting procedure based on the concept of perturbation theory, as showed in Tables 2 and 3, which is consistent with the methodology adopted in ref. 41 for modeling temperature dependent Raman shifts of optical phonons in silicon.^41^

**Table 2:** E^1^_2g_ fitting parameters of the thermal expansion model and multiphonon process model

Location	*ω*_0_	*a*_0_ × 10^−5^	*a*_1_ × 10^−8^	*a*_2_ × 10^−10^	*H*	*F*	Adjusted *R*^2^(E)
Center layer	356.80	0.28 ± 0.20	−6.92 ± 2.40	2.79 ± 0.52	−0.59 ± 0.009	0	0.988
Inter layer	356.78	−1.04 ± 0.14	0.88 ± 1.77	0.74 ± 0.40	−1.21 ± 0.008	0	0.984
Edge layer	356.97	4.84 ± 0.15	20.19 ± 2.13	5.26 ± 0.54	2.25 ± 0.009	0	0.983

**Table 3:** A_1g_ fitting parameters of the thermal expansion model and multiphonon process model

Location	*ω*_0_	*a*_0_ × 10^−5^	*a*_1_ × 10^−8^	*a*_2_ × 10^−10^	*H*	*F*	Adjusted *R*^2^(A)
Center layer	421.73	−3.09 ± 0.25	1.19 ± 3.01	2.38 ± 0.72	−2.17 ± 0.005	0	0.982
Inter layer	421.96	−7.93 ± 0.35	28.06 ± 4.55	−4.42 ± 1.08	−4.04 ± 0.005	0	0.952
Edge layer	421.15	6.98 ± 0.09	57.32 ± 1.73	17.26 ± 0.55	1.36 ± 0.00005	0	0.997

In addition, the uncertainties of *H* and the parameters (*a*_0_, *a*_1_, *a*_2_) were obtained by fixing one quantity at its mean value and then performing the fitting.^38^ It should be noted that, in the thermal expansion model, the uncertainties of (*a*_0_, *a*_1_, *a*_2_) are comparable to their mean values. For certain parameters, such as the *a*_1_ coefficient of the interlayer E^1^_2g_ mode, the uncertainty even exceeds the mean value (1.77 × 10^−8^ > 0.88 × 10^−8^). To evaluate the extent to which parameter uncertainty influences the fitting results, we introduce a deviation coefficient *I*, defined in analogy to the relative error. This coefficient characterizes the relative effect of parameter variations within their confidence intervals on the model outcome, and is expressed as follows:8
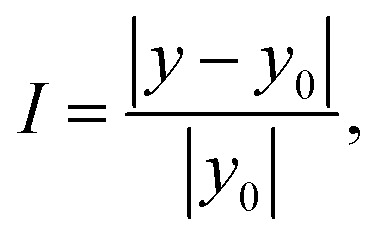
where *y* denotes the model output obtained by randomly selecting parameter values within their confidence intervals, and *y*_0_ represents the model output corresponding to the mean parameter values. A smaller *I* indicates that the fitted model is relatively more precise. Since the fitting results of the three-phonon process are highly precise, with relative uncertainties at least three orders of magnitude smaller than the mean values, *i.e.*, 
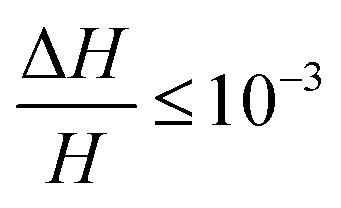
, here *y* and *y*_0_ specifically correspond to the thermal expansion model or the thermal expansion coefficient.

It should be noted that both *a*_0_ and *a*_2_ yield satisfactory fitting results, with uncertainties approximately one order of magnitude smaller than their mean values, *i.e.*, 
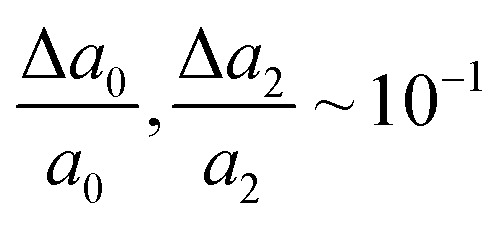
. Therefore, for convenience, their mean values are adopted in the subsequent analysis, while the primary focus is placed on the variation of *a*_1_ within its confidence interval. The calculated results about thermal expansion model are summarized in Table 4, which presents representative *I* values at selected temperatures (150 K, 350 K, and 450 K). As shown, the E^1^_2g_ mode at the center layer exhibits a relatively large *I* (= 5.81) at low temperature. However, this apparent uncertainty arises from the fact that the model value near low temperature approaches zero when evaluated at the mean parameters (as shown in Fig. 5(a)), making its influence (<0.1 cm^−1^) on the overall variation (∼2 cm^−1^) of the model across the range of 150–450 K negligible and thus acceptable. For the other layers, most *I* values remain small (<0.3), confirming the reliability of the parameter fitting. In contrast, a few cases (*e.g.*, the inter layer E^1^_2g_ mode and the center layer A_1g_ mode at high temperature) display larger *I* values (>0.5), inducing that the trend of the corresponding high temperatures is speculative.

**Table 4:** *I* values of the thermal expansion model at different layers

*I*	150 K	350 K	450 K
E center	5.81	2.02	0.89
E inter	0.14	0.52	1.14
E edge	0.02	0.03	0.03
A center	0.07	0.28	0.54
A inter	0.05	0.16	0.21
A edge	0.01	0.01	0.01

We now examine the curves derived from the fitted model. For E^1^_2g_ mode, the observed nonlinear behavior in the curves of the center and edge layers originates primarily from the thermal expansion process as showed in Fig. 5(a) and (c). The redshift of the Raman shift in the center layer results from the combined positive contributions of the three-phonon process and the thermal expansion process, whereas in the edge layer it is mainly influenced by thermal expansion. In the inter layer, a relatively weak nonlinear effect arises from thermal expansion, and the Raman shift variation is principally described by the three-phonon process.

For A_1g_ mode, for the center and edge layers, *i.e.*, Fig. 5(d) and (f), similar to the E^1^_2g_ mode, the nonlinear effect is dominated by the thermal expansion process. The dominant mechanism governing the variation of the Raman shift also differs: in the center layer it is primarily governed by the three-phonon process, whereas in the edge layer it is dominated by the thermal expansion process (as shown in Fig. 5(d) and (f), the red shifts of the A_1g_ mode Raman shifts in the two layers are respectively attributed to the red shifts caused by the positive effects of the three-phonon process and the thermal expansion process). For the inter layer, its nonlinear effect is not pronounced. As the temperature increases, the competing contributions from the three-phonon process and thermal expansion become more pronounced. The redshift caused by the three-phonon process continues to dominate, occurring at a faster rate than the blueshift driven by thermal expansion.

The fitting results provide the relative contributions of the pure temperature effect and the volume effect to the Raman shifts of the two modes at different temperatures. It can be seen that for the edge layers, the absence of outer spirals to constrain the inner spiral volume, combined with relatively weaker interlayer coupling, makes both vibrational modes particularly susceptible to thermal expansion. However, it should be emphasized that no clear distinction between the two spiral domains can be identified from this analysis. The reason lies in the fact that the differences observed in Fig. 4 are mainly manifested in the degree of nonlinearity, whereas the thermal expansion model does not exclusively describe nonlinear effects; rather, it also incorporates the linear redshift jointly determined by the three-phonon process.

To purely characterize the nonlinear effect, one could take the second derivative of the thermal expansion model, which completely removes the linear contribution. In practice, however, the first derivative is sufficient, as it corresponds to dΔ*ω*_V_ = −*nγα*Δ*ω*_V_d*T*. In this expression, the exponential term of e in Δ*ω*_V_ is on the order of 10^−5^ and thus approximately equal to unity, making it nearly proportional to the thermal expansion coefficient. Therefore, it suffices to investigate the properties of *α*, which simultaneously reflects the nonlinear temperature dependence of both modes, while the linear contribution is solely determined by the constant part of *α*.

**Fig. 6:**
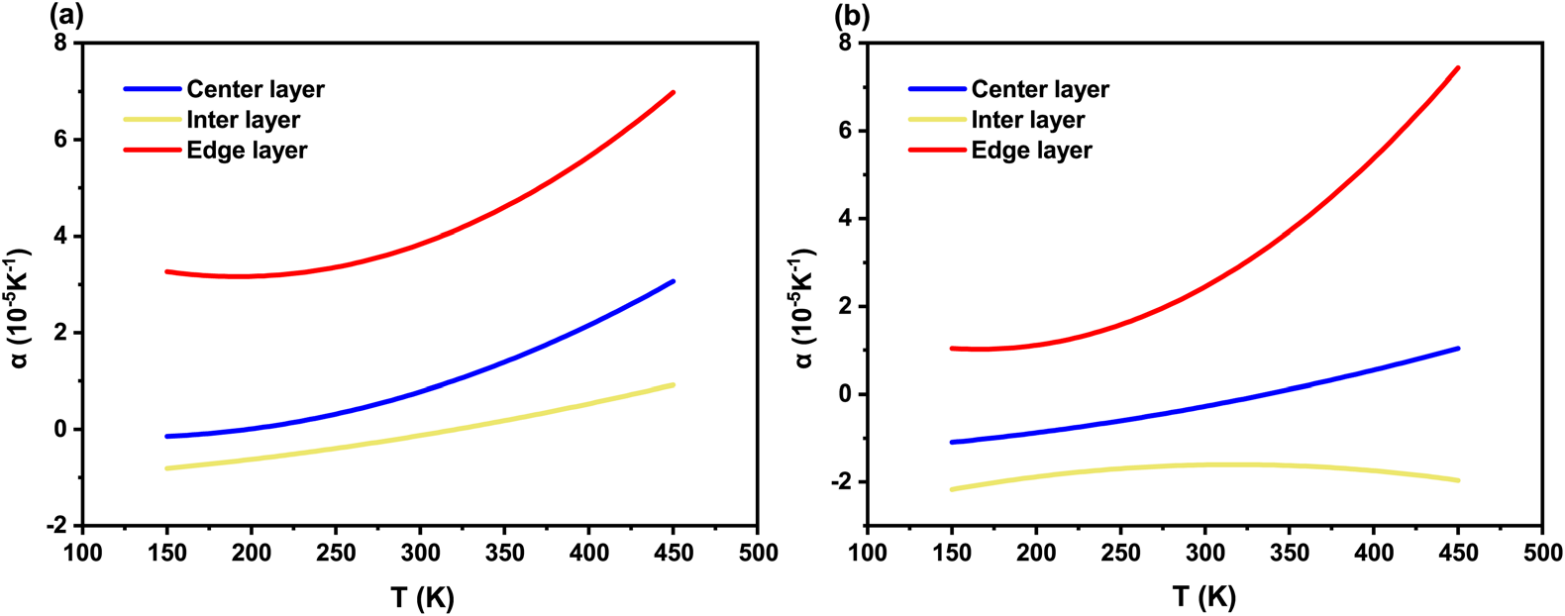
(a) Temperature dependent thermal expansion coefficient curves corresponding to the E^1^_2g_ mode in the outer three layers of the sample. (b) Temperature-dependent thermal expansion coefficient curves corresponding to the A_1g_ mode in the outer three layers. In both plots, the blue solid line represents the center layer, the yellow line corresponds to the inter layer, and the red line represents the edge layer.

To further examine the temperature dependence of *α*, theoretical average values of the Grüneisen parameter, *γ*(E) = 0.9176 and *γ*(A) = 2.1707 (ref. 38 and 42) were used in combination with the fitted parameters of the thermal expansion coefficient listed in Tables 2 and 3. In fact, ref. 42 provides a detailed analysis of the properties of the Grüneisen parameter in WS_2_. Although negative values are observed at certain frequencies, the frequency ranges corresponding to the Raman shifts of the two modes considered in this study exhibit positive values. Moreover, with respect to the temperature dependence of the macroscopic Grüneisen parameter, it remains constant within the temperature range investigated in this work (150–450 K). Therefore, using the average Grüneisen parameter is justified, as it does not affect the interpretation of the curve shape or the identification of key features. Based on this, the temperature dependent curves of the thermal expansion coefficient were obtained, as shown in Fig. 6.

Except for the edge layer, which yields a positive thermal expansion coefficient (>2.5 × 10^−5^ K^−1^ for E^1^_2g_, and > 0.9 × 10^−5^ K^−1^ for A_1g_), all other values exhibit negative coefficients at certain temperature range (≲200 K and ≲350 K for E^1^_2g_ in center and edge layer, ≲400 K and full range for A_1g_ in center and edge layer, respectively). Similar phenomena were also reported in ref. 43 on MoS_2_ and in ref. 44 on monolayer WS_2_. This work presents another potential observation of the negative thermal expansion effect in WS_2_ materials, corroborating the predictions made in ref. 42. However, the relatively large uncertainties in the fitting parameters, particularly at higher temperatures, necessitate a degree of caution. This potential negative thermal expansion effect thus presents an exciting avenue for future confirmation using more precise metrology.

Regarding the shape of the thermal expansion coefficient curves, as the temperature increases, the thermal expansion coefficients associated with both modes generally exhibit an upward trend, except for the A_1g_ mode in the interlayer (its quadratic term *a*_2_ ∼ −4.42 < 0). In other words, in the out-of-plane vibrational direction within 150–450 K, the middle molecular layers contract uniformly with increasing temperature. By comparing the results for each layer, it can be observed that the central and edge layers belonging to the same spiral domain share similar temperature-dependent characteristics of the thermal expansion coefficient. Specifically, under the E^1^_2g_ mode, their temperature-dependent curves are similar, while under the A_1g_ mode they exhibit the same increasing trend, as shown by red and bule line in Fig. 6. In contrast, the interlayer belonging to a different spiral structure shows distinct behavior: under the E^1^_2g_ mode its coefficient varies more smoothly, whereas under the A_1g_ mode it exhibits a convex functional dependence, as shown by yellow line in Fig. 6.

Moreover, It can be observed that the thermal expansion coefficients corresponding to E^1^_2g_ mode are approximately one order of magnitude higher than those predicted by prior experimental and theoretical studies (in which *α*(E^1^_2g_) ∼5 × 10^−6^ K^−1^ for monolayer WS_2_).^38^ Meanwhile, the temperature-dependent curve of the coefficient associated with the E^1^_2g_ mode of spiral WS_2_ differs from that of the monolayer. For the monolayer, the thermal expansion coefficient increases with temperature at a decreasing rate and develops a stationary point around 350 K.^38^ In the present experiment, however, the rate of increase becomes larger with temperature. This implies that for in-plane vibrations, the spiral structure—compared with the monolayer—possesses more degrees of freedom, making the sample more flexible and sensitive to volume changes.

In contrast, the coefficient associated with the A_1g_ mode shows a curve shape similar to that of the monolayer (except for the inter layer). This implies that out-of-plane vibrations are less affected by the structural configuration but exhibit relatively higher sensitivity to interlayer differences due to interlayer coupling effects (as shown in Fig. 6, when compared with E^1^_2g_, the thermal expansion coefficient curves of the center and edge layer in the same spiral domain for A_1g_ show significant differences).

At the end of this section, it is necessary to examine the uncertainty associated with the fitting parameters of the thermal expansion coefficient. Therefore, we calculated the deviation coefficient *I* for the thermal expansion coefficient according to eqn (8), and the results are summarized in Table 5. In regions where *I* > 1, the thermal expansion coefficient approaches zero; similar to the thermal expansion model, this behavior is acceptable. However, unlike the model fitting, one cannot directly conclude the existence of negative expansion in these regions. Nevertheless, the occurrence of negative thermal expansion is still supported. In fact, the inter layer A_1g_ mode and the low-temperature E^1^_2g_ mode both confirm this conclusion, since their uncertainties are insufficient to overturn the result (*I* < 1). On the other hand, at higher temperatures the interlayer thermal expansion coefficient exhibits relatively large uncertainties, and its temperature-dependent trend cannot be fully determined from the data. Even so, the conclusion that layers within the same spiral structure exhibit similar behavior while layers belonging to different spirals show distinct properties remains valid, as it is corroborated by both the low-temperature trend and the evidence presented in Fig. 4.

**Table 5:** *I* values of the thermal expansion coefficient at different layers

*I*	150 K	350 K	450 K
E center	2.76	0.71	0.37
E inter	0.36	3.66	0.91
E edge	0.03	0.04	0.04
A center	0.18	5.41	0.6
A inter	0.14	0.38	0.46
A edge	0.01	0.01	0.01

## 4 Conclusions

In conclusion, we have systematically investigated the layer-specific temperature-dependent Raman spectra of a double-spiral WS_2_ structure over a wide temperature range (150–450 K). We find that the complex geometry of a double spiral leads to a distinctly weaker thermal response compared to both monolayer and single-spiral WS_2_. This phenomenon is attributed to the unique interplay between strain induced by the spiral geometry and interlayer coupling, which is modified in the double-spiral architecture. Crucially, we discovered that the thermal behavior is domain-dependent: layers within the same spiral domain exhibit similar responses, while layers from different spirals show discernibly different trends. This result provides unprecedented insight into the phonon physics of these complex nanostructures. Furthermore, by employing a combined thermal expansion and anharmonic phonon model, we elucidated that the nonlinear temperature dependence is predominantly governed by thermal expansion, with the three-phonon process determining the shift magnitudes—except for the edge layer. Notably, the fitting results suggest the potential presence of a negative thermal expansion effect in certain layers and temperature ranges, which aligns with theoretical predictions for WS_2_.^42,44^ This intriguing observation, although requiring further verification with more precise techniques, points to the complex coupling phenomena inherent in the double spiral structure. This work addresses the critical need to understand how complex morphological hierarchies in 2D materials, beyond simple monolayers or twisted layers, influence their fundamental physicochemical properties. The insights gained here into the domain-specific thermal properties of spiral structures are vital for their future application in thermal management, phonon engineering, and flexible optoelectronics. While the limitations of our current setup suggest avenues for more precise future studies, the clear trends and consistent models presented herein firmly establish the unique thermal characteristics of double-spiral WS_2_. We believe our study lays the groundwork for further exploration of defect-engineered and morphologically complex 2D materials.

## Conflicts of interest

There are no conflicts of interest to declare.

## Data Availability

The data supporting this article have been included in the manuscript. Raw data will be provided upon reasonable request.
